# Probabilistic Nucleation and Crystal Growth in Porous Medium: New Insights from Calcium Carbonate Precipitation on Primary and Secondary Substrates

**DOI:** 10.1021/acsomega.1c04147

**Published:** 2021-10-12

**Authors:** Mohammad Nooraiepour, Mohammad Masoudi, Nima Shokri, Helge Hellevang

**Affiliations:** † CO_2_ Storage Research Group, Department of Geosciences, University of Oslo, P.O. Box 1047 Blindern, 0316 Oslo, Norway; ‡ Institute of Geo-Hydroinformatics, Hamburg University of Technology, Am Schwarzenberg-Campus 3 (E), 21073 Hamburg, Germany

## Abstract

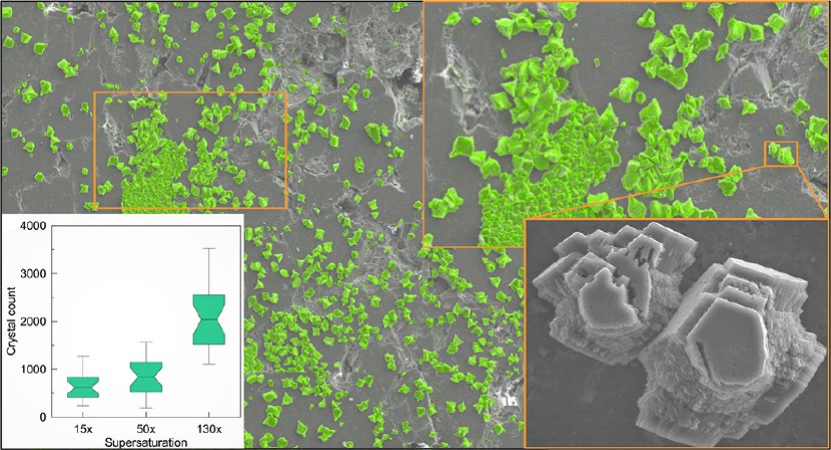

Knowledge of crystal nucleation and growth is paramount in understanding the geometry evolution of porous medium during reactive transport processes in geo-environmental studies. To predict transport properties precisely, it is necessary to delineate both the amount and location of nucleation and precipitation events in the spatiotemporal domain. This study investigates the precipitation of calcium carbonate crystals on a heterogeneous sandstone substrate as a function of chemical supersaturation, temperature, and time. The main objective was to evaluate solid formation under different boundary conditions when the solid–liquid interface plays a key role. New observations were made on the effect of primary and secondary substrates and the role of preferential precipitation locations on the rock surfaces. The results indicate that supersaturation and temperature determine the amount, distribution pattern, and growth rate of crystals. Substrate characteristics governed the nucleation, growth location, and evolution probability across time and space. Moreover, substrate surface properties introduced preferential sites that were occupied and covered with solids first. Our results highlight the complex dynamics induced by substrate surface properties on the spatial and temporal solute distribution, transport, and deposition. We accentuate the great potentials of the probabilistic nucleation model to describe mineral formation in a porous medium during reactive transport.

## 1 Introduction

During solute transport and flow of a reactive fluid within a porous medium, crystal nucleation and growth may change the pore geometry and thus transport properties of the porous medium.^1−4^ Solid precipitation and accumulation also change the available surface area for nucleation and growth, which leads to alterations in the reactivity of the system, reaction rates, and therefore, overall progress.^4−7^ Predicting potential events and the evolution of transport properties of a porous medium is paramount in many natural and engineered systems dealing with reactive transport processes coupled with thermo–hydro–mechanical effects. Fluid flow, solute transport, chemical reactions, and fluid–solid interactions may vary markedly over different time- and length-scales in such systems and processes.^1,8−10^ Prediction of such complex natural or engineered perturbations is far from trivial in geo-environments. It requires an updated understanding of crystal nucleation and growth, governing factors, and interactions at solid–liquid surfaces and interfaces.

A better understanding of nucleation and precipitation facilitates better design and production of crystalline materials, from amorphous to nano-sized crystal assemblages, to large inorganic super-pure crystalline solids for industrial applications.^11−14^ Delineating kinetics and growth dynamics are particularly important for synthesizing materials with functional groups such as proteins^15−17^ or building blocks such as ionic compounds^18−21^ because factors such as crystal size, shape, number, accumulation pattern, and defects are kinetically controlled. Based on classical nucleation theory (CNT), crystal nucleation and growth kinetics of an aqueous solution are governed by supersaturation, temperature, interfacial free energy between the nucleating mineral and substrate, and solution composition. Therefore, interaction among several mechanisms and parameters control the ultimate kinetics of crystal growth.^19,20,22−25^

For ionic compounds such as calcite and gypsum,^19,26^ mineral growth has been explained using the Burton–Cabrera–Frank model^27,28^ with some of the limitations being discussed in other studies.^18,19^ As we previously presented,^11,29^ there is a dependency between the growth rates of ionic minerals (composed of cation and anion units) and the solution stoichiometry. These growth rates for ionic minerals reach the highest point for a fixed aqueous oversaturation when the ionic concentration ratio is equal or close to the stoichiometric [cation]/[anion] activity. Moreover, the aqueous solution stoichiometry controls the advancement rates of growth orientations.^30,31^

Although the traditionally used mineral growth models and CNT may successfully predict the amount of precipitations, they fail to predict where crystals will nucleate and form in the pore space. It is critical to predict the distribution of the crystals besides their amount, particularly when geometry evolution in porous medium and consequent clogging is expected.^1,5,32−35^ Understanding how the dynamics of fluid flow and solute transport may be modified in geo-environmental applications requires knowledge of the individual and collective behavior of mineral nucleation and growth under different boundary conditions. Different boundary conditions affect the changes in porosity and permeability of porous media induced by the crystallization process. Nucleation is the pre-growth process governing the primary position of crystal formation and subsequent growth. Mineral nucleation is a probabilistic process, where crystals can nucleate anywhere under similar conditions such as surface properties, supersaturation, and temperature. Therefore, it is imperative to use a probabilistic approach or an upscaled physically sound representation to understand the effect of mineral nucleation on the dynamics of fluid flow and solute transport in porous media.^7^

Motivated by the importance of crystallization and growth kinetics in a variety of multiphase and multiscale processes occurring in geo-environmental processes and systems, this paper extends the understanding of factors controlling crystal nucleation and growth rates, the impact of ambient and aqueous phase properties, and the substrate characteristics. The surface mineral synthesis experiments were performed with a threefold purpose: (a) to provide new insights into probabilistic crystal nucleation and growth, (b) to delineate how primary and secondary substrates govern solid accumulation, and (c) to describe heterogeneous nucleation as a function of aqueous-phase supersaturation, temperature, and evolution time. We use laboratory experiments and high-resolution surface imaging techniques to show the importance of the spatial and temporal location and the distribution of nucleation and growth events, particularly when the interplay among several determining parameters is inevitable. To explore the effect of solute concentration, temperature, and experimental elapsed time on the surface coverage area and the number of precipitated crystals, we carried out calcium carbonate synthesis experiments on the surface of heterogeneous quartz-rich sandstone with a solution stoichiometry of close to 1 (*C*_Ca_/*C*_CO_3__ ≈ 1).

## 2 Results and Discussion

### 2.1 Supersaturation and Temperature Control on Crystal Precipitation

Analyses of Brumunddal sandstone substrates show a texturally mature arkosic to subarkosic arenite with a mineralogical composition of 72–81% quartz, 16–23% feldspar, 1–2% mafic fragments, and approximately 2–5% carbonate cement and clay minerals. Therefore, the substrates represent a natural porous medium mainly comprising quartz, feldspar, and intergranular carbonate cement.

**Figure 1:**
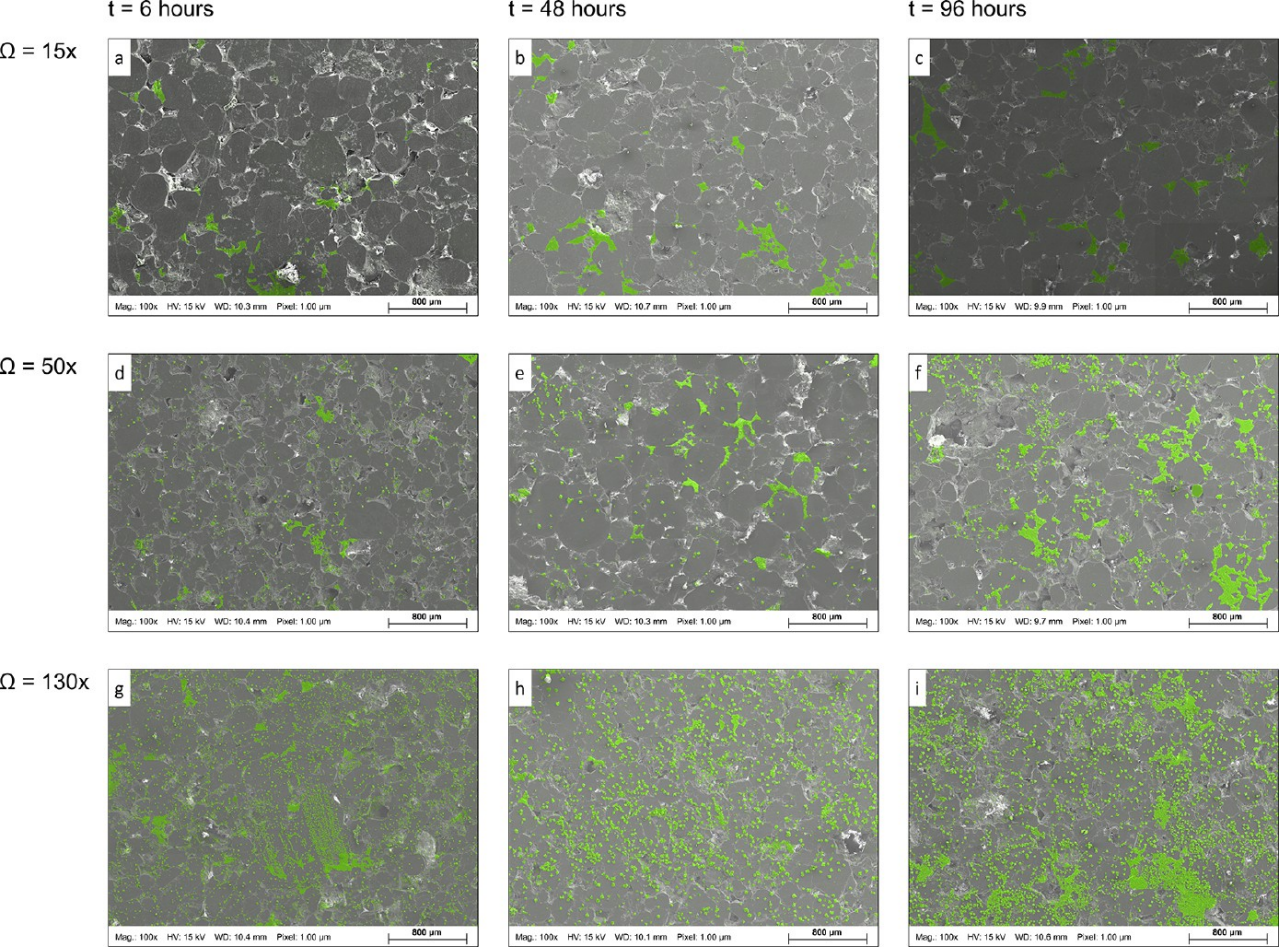
Surface maps of heterogeneous sandstone substrates at different supersaturations (Ω) and elapsed times from the onset of the experiment (*t*) for the laboratory tests conducted at temperature *T* = 60 °C. Top: Ω = 15 (a–c), middle: Ω = 50 (d–f), and bottom: 130 (g–i).The mosaic maps demonstrate the superimposed EDS on top of the BSE SEM to identify calcium carbonate crystals (color-coded in green). Each mosaic map consists of nine BSE SEM images (3 × 3) covering approximately 10.5 mm^2^, with a spatial resolution of 1 μm. Magnification: 100*×*.

Figure 1 presents the energy-dispersive X-ray spectroscopy–scanning electron microscopy (EDS–SEM) surface maps of nine experiments conducted at *T* = 60 °C. Each row shows a given supersaturation (Ω = *Q*/*K* = 15, 50, and 130*×*), and each column indicates a similar elapsed time (6, 48, and 96 h). The mosaic maps show calcium carbonate crystals (color-coded in green) on top of the backscatter SEM micrographs. Two trends are evident in Figure 1. First, an increase in Ω and extended *t* contributed to an increase in the number and the total coverage area of crystals. Second, the patch sizes (connected precipitates) also increase under higher Ω–*t* conditions (e.g., Figure 1i compared to Figure 1a). The impact of extended elapsed time from the onset of the experiment is pronounced for higher supersaturations, particularly for Ω = 130, owing to the continued and available solute concentration in the substrate’s proximity within the aqueous phase. Comparison between the different supersaturations suggests that at lower Ω values, a more selective pattern for the location of crystal nucleation and growth can be identified. At high supersaturations (Ω = 130, Figure 1g–i), an overall closely packed pattern is observable where crystals tend to cover the surface of the substrate more uniformly.

**Figure 2:**
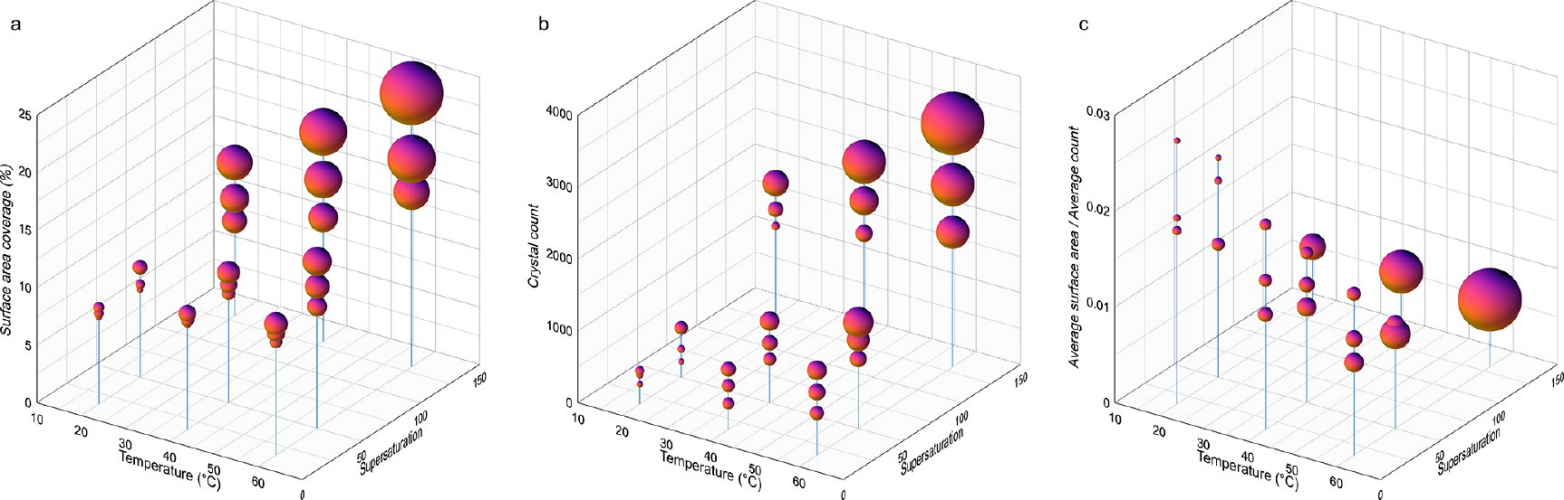
(a) Total surface area coverage (%), (b) number of the precipitated crystals, and (c) ratio of average surface area coverage to the crystal count number on *z*-axis is plotted as a function of the pair of temperature and supersaturation on abscissa and ordinate (*x*–*y* plane). The data points are derived from image processing of three random locations within each mosaic map and represent three supersaturations (Ω = 15, 50, and 130*×*) and three temperatures (*T* = 20, 40, and 60 °C). The size of balls in the subplots illustrates average count (a) and average surface area (b,c).

The image processing results of 27 experiments (3 × 3 × 3 sets of Ω–*T*–*t*) are presented in Figure 2, where the total surface area coverage (%) and crystal count on the *z*-axis are plotted versus the pair of temperature and supersaturation on abscissa and ordinate. Figure 2c shows the ratio of average surface area coverage (%) divided by the crystal count number on the *z*-axis. The size of balls in the subplots illustrates the average count (Figure 2a) and average surface area (Figure 2b,c). The results are also shown as a function of elapsed time from the onset of the experiment in Figure S4 (Supporting Information). Figure S4 also shows the computed entropy (*E*) of each mosaic map on the second ordinate (*y*-axis), quantifying randomness within the system. In Figure 2, with the increase in supersaturation, the distinction between the data points representing different surface areas and crystal counts becomes more visible. The crystal coverage area and count increased remarkably for higher Ω values, particularly for Ω = 130 (Figure 2a,b). The increase in the coverage area and crystal counts over time for a given Ω are subtle for *T* = 20 °C compared to elevated *T*_s_ (Figure S4). Additionally, at low supersaturation (Ω = 15) for all three *T*_s_, the number of new crystals compared to that at *t* = 6 h shows a limited increase for the rest of the experiments (*t* = 48 and 96 h) (Figure S4a,b). The area to count ratio in Figure 2c shows a decline with the increase in temperature and supersaturation. The scatter in data points under a given condition (fixed *T*–Ω) also decreased at higher *T*–Ω values (Figure 2c). The lower area to count ratio can be associated with the higher number of precipitated crystals at higher *T*–Ω values that did not coalesce and interconnected patches with comparable areal coverage.

At lower supersaturations, the probability of nucleation is lower. As a result, fewer numbers of stable nuclei form at an early time. These stable nuclei consume the available solute in the solution to grow, decreasing the probability of new nucleation events. Thus, low to moderate supersaturations bring about more connected and more extensive solid accumulations. However, when the saturation ratio is too high (e.g., Ω = 130), the driving force for growth and nucleation is significant and scattered patterns might also form. In other words, the low-Ω case is associated with the limited solute concentration within the stock solution, limiting the number of new nucleation events and therefore less support of the continued growth of previously precipitated solids at early times. At higher supersaturations (Ω = 50 and 130), enough solute is present in the solution to support crystal continued nucleation and growth.

As indicated in Figure S4a,c,e on the second *y*-axis, when the number of precipitated crystals and areal coverage increases, entropy quantifying randomness and disorder within each surface map also shows an increase. At the beginning of experiments, an ordered system exists where no crystal is present on the surface. The entropy (*E*) then increases until it reaches a maximum value, approaching one for an entirely random system. We expect the measure of randomness to decline afterward when crystals start covering more and more surface area, and the entropy eventually reaches a constant minimum value if the substrate is fully covered.^7^ The higher number of crystals and larger surface coverage are translated into greater Shannon entropy values. The thermodynamic entropy and entropy defined in information theory capture increasing randomness within the system.^46,47^ The larger computed entropy reflects higher disorder within the system, which is the available substrate for crystal nucleation and growth. When only limited solute concentration is available for crystallization and deposition processes (e.g., Figure S4a,b where Ω = 15), the degree of disorder increases due to mineral nucleation. In such cases, the growth is insubstantial and changes slightly over time. However, the availability of a high concentration of a supersaturated aqueous phase supports continued crystal nucleation and growth on the primary and secondary substrates. Hence, it contributes to a notable increase in system randomness from ordered initial states with *E* = 0. As Figure S4 indicates, for Ω = 50 and 130, the increase in entropy values are observed until *E* = 0.4 and 0.65, respectively. The derivative of changes suggests a half Gauss–Laplacian entropy evolution.

**Figure 3:**
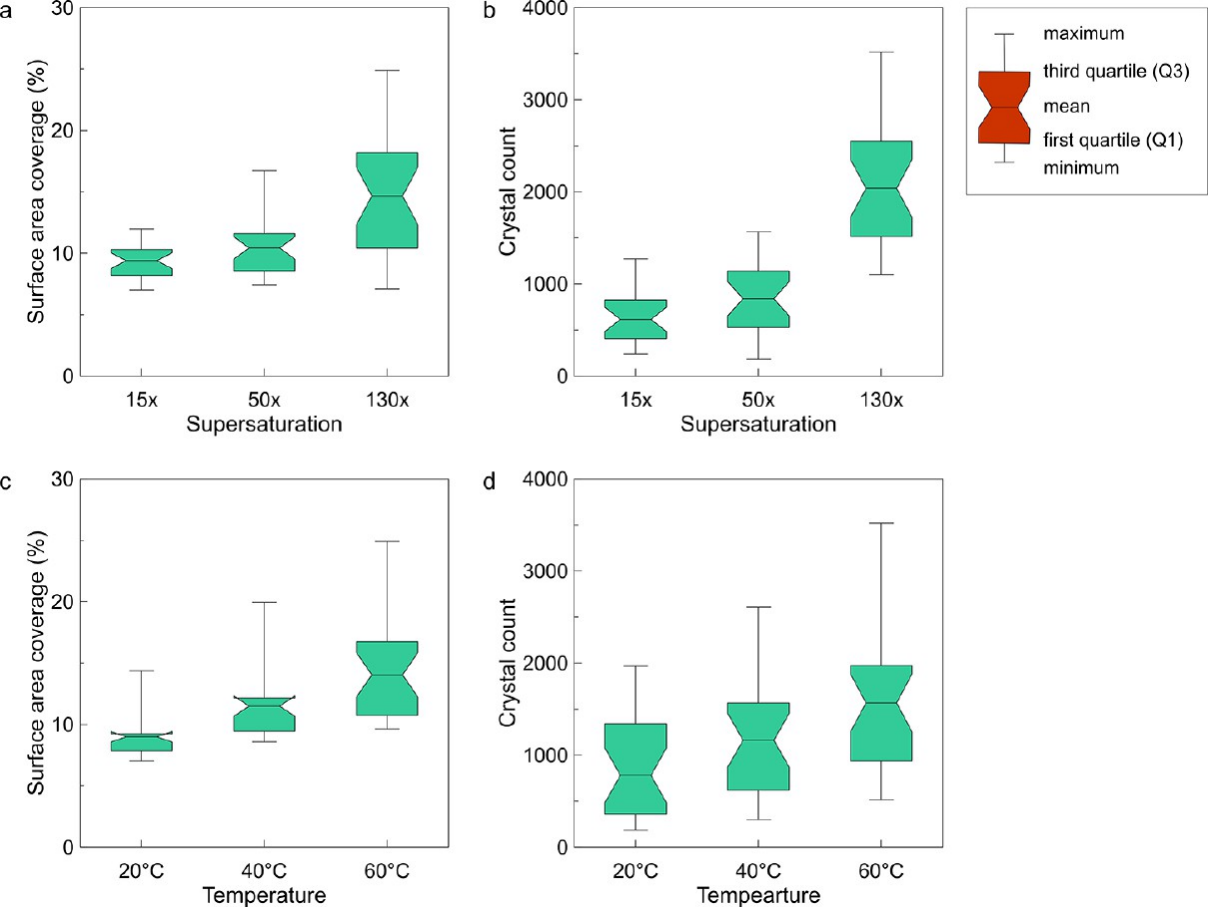
Box plots of total surface area coverage (%) and the number of precipitated crystals vs (top, a,b) supersaturation and (bottom, c,d) temperature, obtained by image analysis along with the skewness, quartiles, and mean value. The whiskers indicate variability outside the upper and lower quartiles (25 and 75%) and represent the minimum and maximum values.

The boxplots in Figure 3 show the distribution of the numerical data along with the skewness, quartiles (percentiles), and mean values. The whiskers (lines extending from the box plots) indicate variability outside the upper and lower quartiles and represent the minimum and maximum values. In Figure 3, the surface area coverage (%) and crystal count are plotted against the supersaturation and temperature.

Figure 3 demonstrates the significant effects of solute supersaturation and temperature on crystal nucleation and growth. Comparison of mean values indicates that with an increase in supersaturation, a semi-exponential growth (*y* = *a·*e^*b*^*·*Ω, where *b* ≤ 0.01) and with an increase in experimental temperature, a semi-linear growth (*y* = *a*·*T* + *b*) in the surface area coverage and the number of crystals can be identified. The distance between minimum and maximum (variability domain) is also increased with the increase in Ω and *T*. In other words, as the entropy increases, the degree of disorder within the system increases, and as a result, more scattered and broader distribution functions are expected for the nucleation and growth events. Comparison between the effect of Ω and *T* (top and bottom row subfigures) shows that the expected variability and scatter at a given *T* is more significant than at a given Ω. It highlights the effect of temperature on the randomness of crystal formation and deposition patterns and more considerable variations that one may expect when temperature changes compared to supersaturation. In summary, our results suggest that temperature has a significant effect on nucleation (especially when compared to effects of supersaturation), and because nucleation is probabilistic, the variability driven by the temperature changes is more pronounced.

### 2.2 Nucleation and Growth Patterns

As it is shown in Figures 1, S1, and S3, stochastic yet selective spatial location for crystal nucleation and growth is evident. The crystals are randomly distributed over the substrate surface with several preferential locations where most interconnection and collective growth occurred. We will further discuss this phenomenon in the next section.

Another insight from Figures 1, S1, and S3 is the notable consumption of solute concentration in the vicinity of growth sites. For instance, in Figure 1f, limited or no visible crystal growth happened near the large solid accumulations. Simultaneously, the number of precipitated crystals is significantly higher in the regions with isolated crystals or smaller patches of calcium carbonate. It indicates that large solid accumulations can attract and consume more supersaturated fluid in their peripheral region. According to the probabilistic nucleation model,^7^ there is a similar probability for nucleation at various sites on the substrate, under similar conditions (*T* and Ω). However, the nuclei may not find the opportunity for growth and realization as stable isolated crystals when there is insufficient solute available to support continued growth. Additionally, the occurrence of a nucleation event depends also on the saturation ratio. Hence, the lower the saturation ratio, the lower the chance of nucleation.

The solute consumption makes the peripheral regions devoid of the supersaturated solution and hence less favorable for nucleation and growth. Consumption of supersaturated solution has more influence at lower Ω (Figure 1c) than at the higher Ω values (Figure 1i). Devoiding the supersaturated solution is controlled by the interaction between the reaction rate and the mode of mass transport (advection and diffusion; here, only diffusion). When the growth rate is large enough and the solute transport cannot compensate for the consumed solute, the impact of devoiding in the peripheral regions is more significant. In Figure 1i (Ω = 130), the solid accumulation affected the area in the bottom right side of the subfigure considerably, owing to the effect mentioned in the previous paragraph, although enough solute concentration is present in the aqueous phase enabling continued and uninterrupted growth. In contrast, the top left side hosts a higher number of isolated or minor patches.

**Figure 4:**
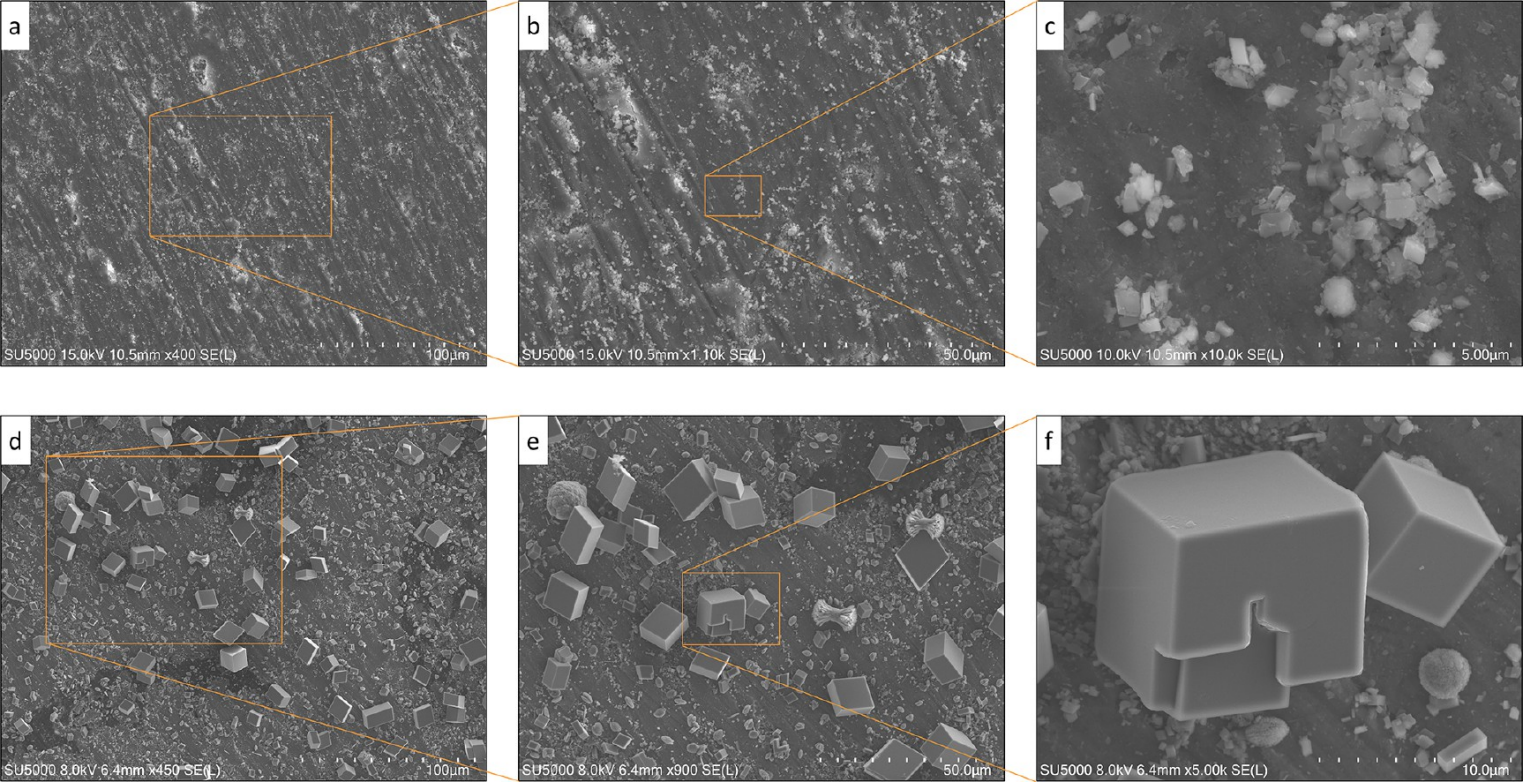
Effect of growth time on the magnitude, distribution, and growth of calcium carbonate crystals after the induction of nuclei. SEM surface maps (SE imaging technique) show experiments with similar supersaturation (Ω = 130) but different elapsed times from the onset of the experiment, (top, a–c) *t* = 3 h, and (bottom, d–f) *t* = 48 h. In each row, magnified SEM images are presented sequentially down to the crystal level. Magnification for each subfigure is as follows, respectively: (top) 400, 1100, and 10,000*×* and (bottom) 450, 900, and 5000*×*.

The secondary electron (SE) microscopy was used to investigate the impact of growth time on the magnitude, distribution, and growth of calcium carbonate crystals. Figure 4 demonstrates typical images recorded by SE microscopy of the samples with similar supersaturation levels (Ω = 130) but different elapsed times. The top and bottom row represents 3 and 48 h elapsed time from the onset of the experiment. In each row, magnified SEM images are presented sequentially down to the crystal level. Figure 6 also shows different stages of crystal formation, from primitive non-crystalline geometries to ordered rhombohedral calcium carbonate crystals.

Figure 4a shows the substrate surface covered with initial-state crystal forms. Relatively uniform surface coverage exhibits similar physiochemical conditions in the portrayed substrate region that supported stochastic but compact nucleation sites. The enlarged regions provide evidence for the probabilistic spatial distribution of nuclei and crystals, as addressed in our newly developed probabilistic nucleation model.^7^ The top-row subfigures suggest that 3 h elapsed time from the onset of the experiment was not long enough to transform a nucleus to the primitive form and subsequently to the geometrical crystalline form (Figure 4c). The coalescence and interconnection of the precipitates occurred even in the early stage of the experiment (Figure 4b,c). In contrast to the early-phase evolution in the top row, the bottom row demonstrates the developed crystalline calcium carbonates of different sizes, up to 15 μm (Figure 4d–f). As shown in Figure 4d, the substrate is covered with primitive and crystalline forms of different shapes and sizes. As previously discussed, consumption and voiding of solute concentration occurs in the vicinity of larger crystals and solid accumulations. This phenomenon is depicted in Figure 4e, where the growth for the other less prominent solids is restricted near the coalesced large crystals. The number of primitive and crystalline solids is also limited in such regions. The affinity to grow collectively to form one connected crystalline mass might arise from the tendency to attract more supersaturated fluid and facilitate continued nucleation and growth.

In Figure 4d–f, rhombohedral, prismatic, and semi-tabular calcite crystals can be identified. The primitive rhombohedron (r {100}), flatter rhombohedron (e {110}), and a more acute one (f {111}) are presented in Figure 4e. In Figure 4e,f, prismatic habits as a combination of the prism and rhombohedron and tabular-form crystals can be observed. The imperfect crystal geometries, for instance, single isolated calcite crystals in Figure 4f, are due to impurities in the aqueous phase, which can be traced back to dissolved salts used to make the stock solutions or some dissolutions from the substrate during the experimental time. The solute impurities contributed to structural defects in the rhombohedral calcium carbonate crystals.

### 2.3 Effect of Primary and Secondary Substrates

This section provides visual evidence of high-resolution SEM of mineral nucleation and crystal growth with a threefold purpose. We delineate mineral precipitation on (a) secondary substrates (previously precipitated crystals), (b) primary substrates when carbonate cement is present (nearly similar composition in the substrate and the aqueous phase), and (c) multi-mineral heterogeneous substrates and potential preferential sites. For part (c), we present surface and elemental phase maps provided by the EDS.

**Figure 5:**
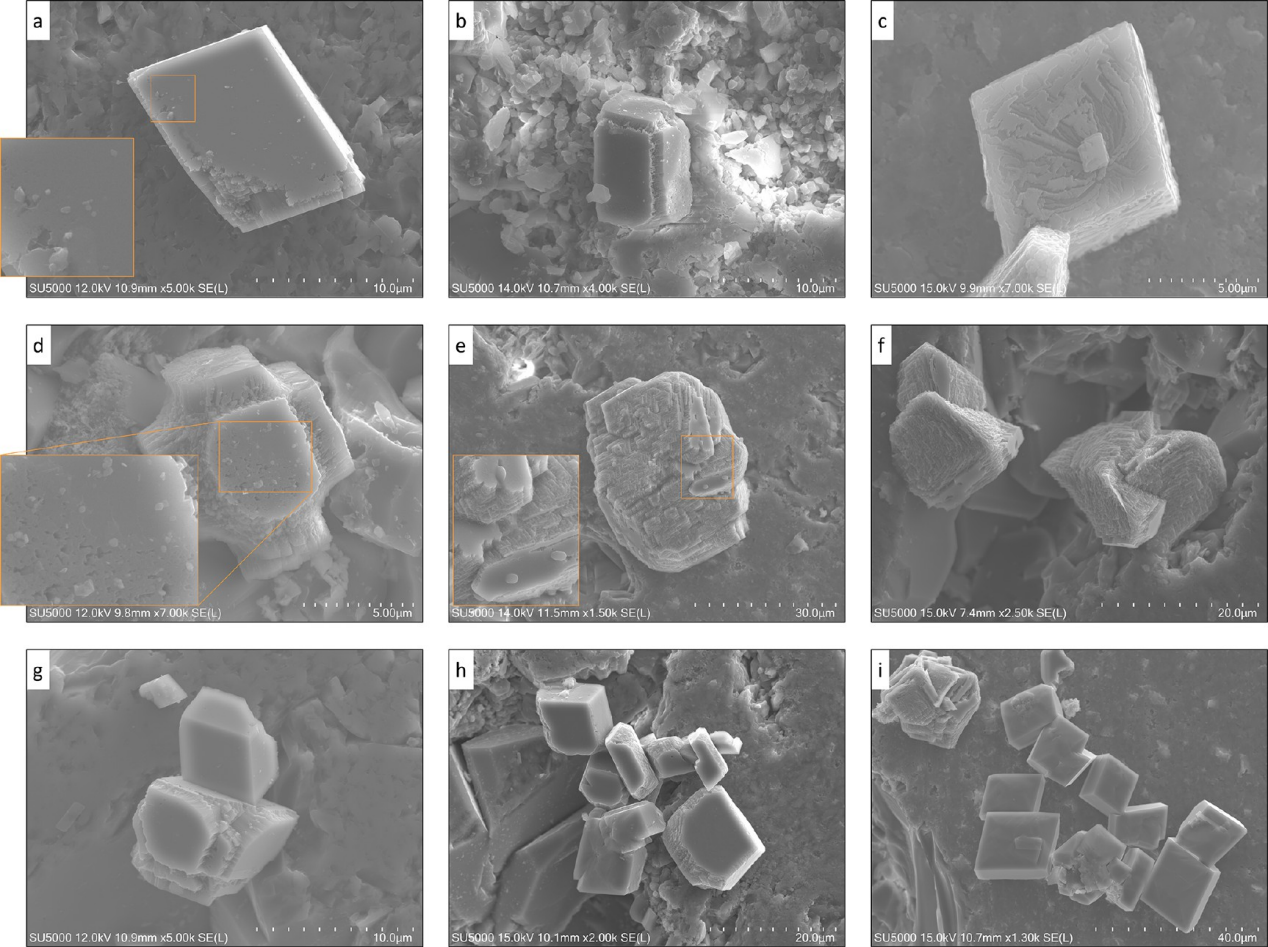
Evidence of crystal nucleation and growth on previously precipitated crystal surfaces, named secondary substrate in the article (a–i). In the enlarged regions in subfigures (a,d,e), nucleation and formation of primitive forms on solid surfaces are shown, down to sub-micron resolution. Magnification and length scale of the SEM surface maps (SE imaging) are given for each subfigure.

In Figure 5, evidence of crystal nucleation and growth on previously precipitated crystals is presented. In the enlarged regions (Figure 5a,d,e), precipitation of primitive forms on crystal surfaces are shown, down to sub-micron resolution. For reactive transport modeling (RTM) of mineral nucleation and growth, one may hypothesize that the ratio between the reaction rate and nucleation rate solely controls the growth pattern and can be used as a proxy for predicting geometry evolution in porous medium across scales. However, present results refute the hypothesis because besides fluid–solid physicochemical conditions, there is a competition between the primary and secondary substrates to attract the supersaturated solution and facilitate new nucleation events. The affinity for nucleation and growth of secondary minerals is higher adjacent or on top of the newly formed crystals than on the original foreign substrate (Figure 5). The formation of the first nucleus on the substrate creates a secondary substrate with a higher potential (probability) to form the following nuclei.^33^ It incorporates another layer of complexity into the RTM, which is necessary to be taken into account to capture the reality of nucleation and crystal growth. This dimension is realized and integrated into the probabilistic nucleation model described by Nooraiepour et al.^7^

In other words, the marked difference between the interfacial free energy of the primary and secondary substrate determines the probability and affinity of new nucleation events. Furthermore, the preferential affinity to nucleate and grow on top and adjacent to secondary substrates also contributes to increased coalescence between the crystals (e.g., Figure 5i). It, in turn, will facilitate attraction of increasingly more solutes toward the solid accumulation sites and therefore a faster growth rate compared to areas with isolated and dispersed crystals. Such coalesced solid patches are of paramount importance as they significantly alter pore network geometry, particularly throat sizes, and hence transport properties affecting fluid flow and solute transport pathways within the porous medium.

**Figure 6:**
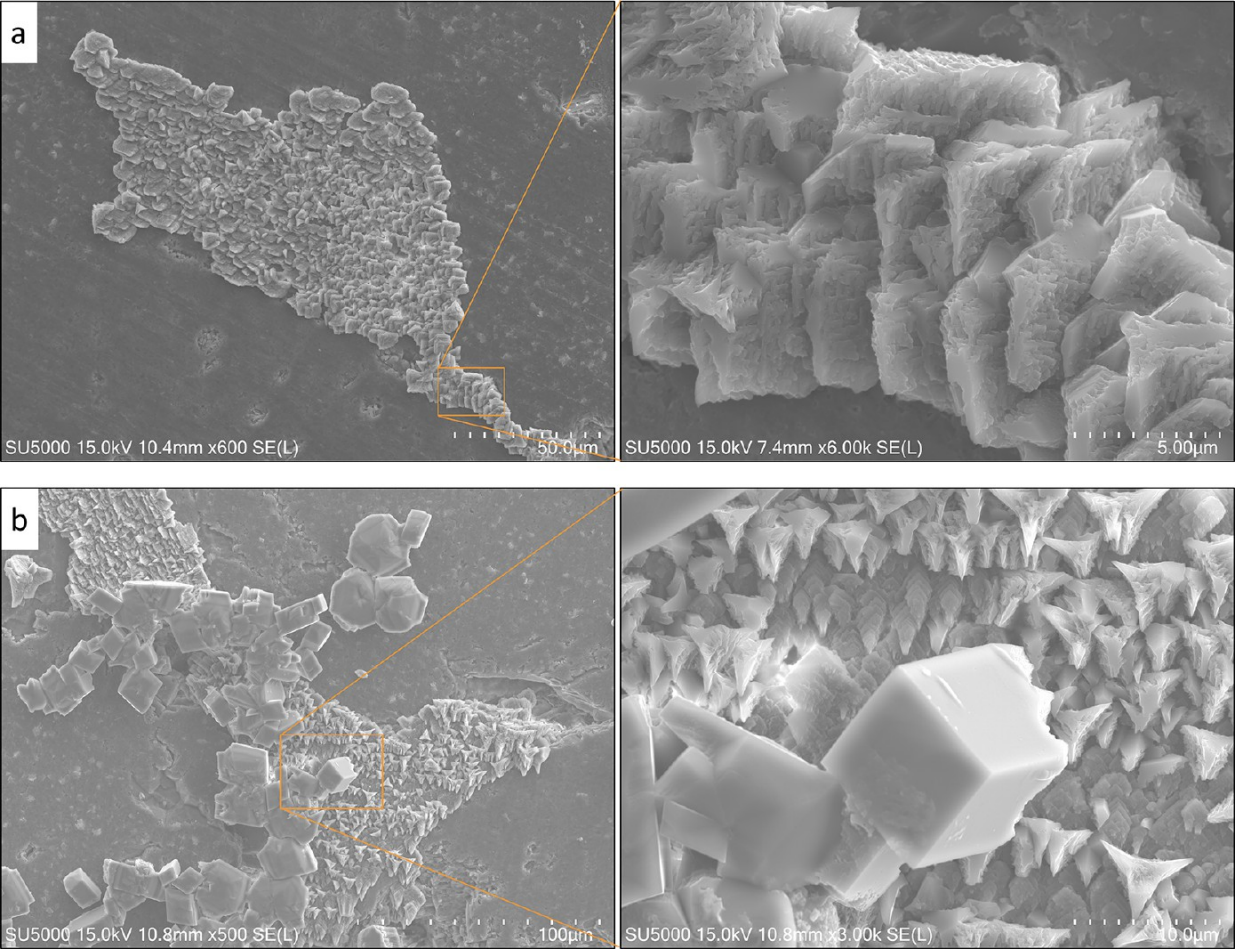
SE surface maps of calcium carbonate precipitation on the heterogeneous sandstone substrate, where intergranular carbonate cement is present. (a) Semi-conform growth patterns and (b) isolated crystalline mineral formation are illustrated in the enlarged sections of subfigures (a,b). Magnification and length scale are given for each subfigure.

Figure 6 shows SE micrographs of the substrate, where carbonate cement is present. In each subfigure, SE surface maps are enlarged to visualize how mineral precipitation occurred on top of the intergranular carbonate cement. As shown in Figure 6a,b, there are almost no growth events on the quartz-rich sandstone substrate near the carbonate cement. The available solute concentration for nucleation and precipitation is attracted and consumed by the previous carbonate patches (intergranular cement), owing to favorable interfacial free energy. The morphology of accumulated crystals is also markedly different from what is shown in the previous figures. For instance, in Figure 6a, a semi-conform precipitation and growth pattern is illustrated in which individual crystals show a compact, coalesced, and overgrowth pattern. The co-presence of semi-conform precipitation patterns with coalesced crystalline geometries is also presented (Figure 6b).

Figure 6 suggests that even on carbonate cement and semi-conform accumulations, there is still a high probability of nucleating and growing crystalline geometries (Figure 6b). Such a phenomenon will exceptionally be critical when describing the effects of crystallization-driven clogging in reactive transport models. The three-dimensional structure and morphology of these crystals could modify or even block the pore structure and pathways affecting the subsequent flow and transport process. Without such information, our description of the effects of solute crystallization in porous materials would primarily rely on empirical parameters without adequate consideration of the actual physics governing the process.

**Figure 7:**
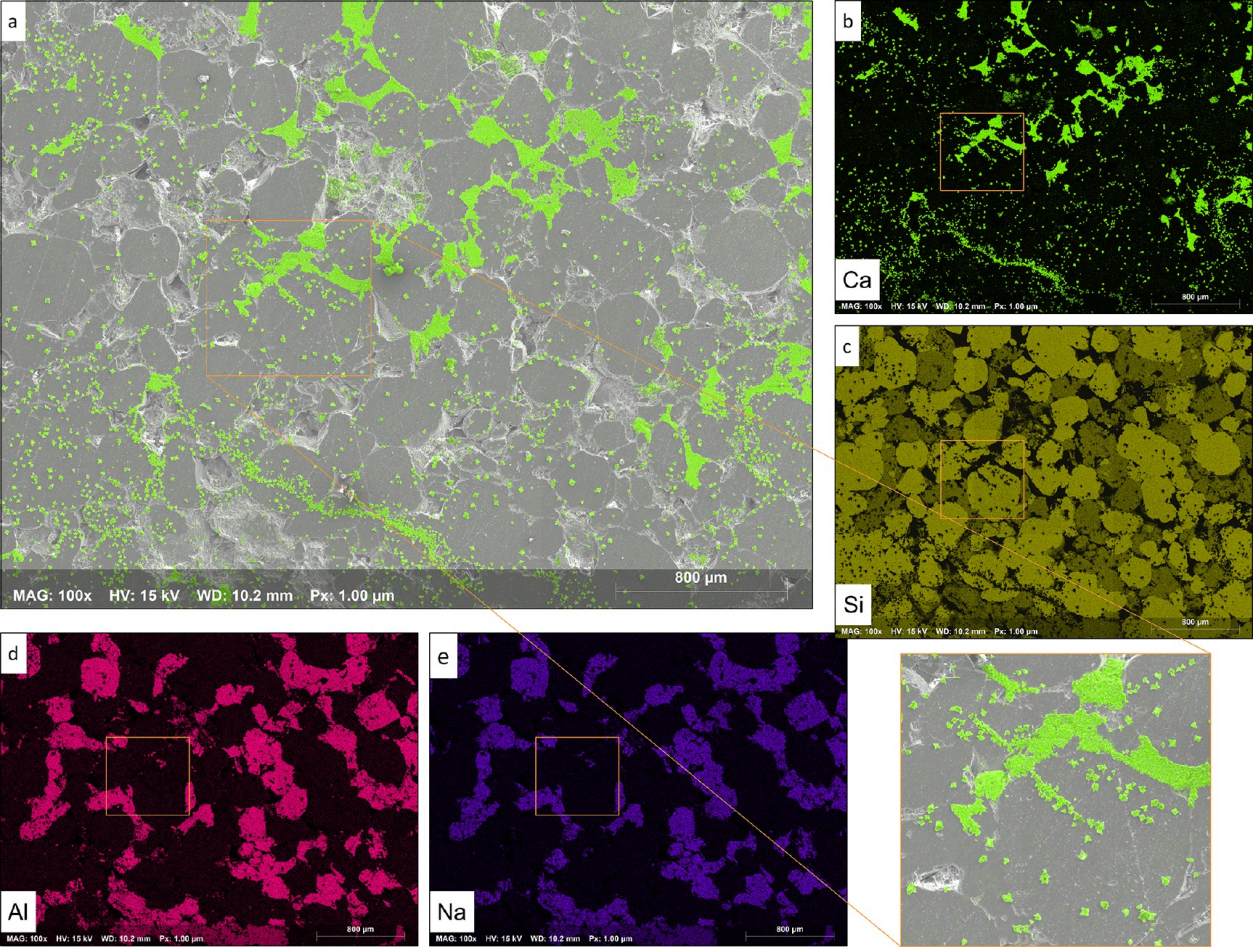
EDS elemental phase maps of mineral formation on a multi-mineral heterogeneous substrate and preferential affinity for nucleation and growth. (a) SEM–EDS surface map with calcium carbonate crystals color-coded in green, a magnified section of the mosaic map, along with elemental phase maps of (b) calcium (Ca) representing calcite, (c) silica (Si) representing quartz, and (d) aluminum (Al) and (e) sodium (Na) both representing feldspar grains. Magnification = 100*×* and length scale = 800 μm.

Finally, the EDS elemental phase maps presented in Figure 7 provide insight into the solid formation on a multi-mineral heterogeneous substrate and preferential affinity for nucleation and growth. In Figure 7, we present an SEM–EDS mosaic surface map with calcium carbonate crystals color-coded in green, a magnified section of the substrate, along with elemental phase maps of calcium (Ca) representing calcite, silica (Si) representing quartz, and aluminum (Al) and sodium (Na) representing feldspar grains. We will focus on the preferential affinity toward quartz or feldspar and identify favorable locations chosen first for nucleation and crystallization.

Detailed investigation of high-resolution EDS–SEM surface maps indicates that at lower supersaturation (Ω = 15), more selective nucleation and growth phenomena occur, where spots with more favorable surface characteristics will be selected first. At higher Ω values (Ω = 130), visual randomness and entropy are apparent. Even though a highly disordered system in Figure 7a is demonstrated where crystal growth is detectable almost everywhere, a detailed evaluation shows that several favored spots are still among the preferred locations. The favored sites for nucleation and subsequent growth are in the following order:

- carbonate cement between the sandstone grains, owing to the considerably lower interfacial free energy between the precipitating phase and carbonate cement compared to other grain-forming minerals,
- hollow and indentation surfaces such as holes and rough regions, and
- around the grain edges, which can also be related to irregularity and roughness of the substrate among the grain edges providing preferential sites for crystallization compared to interior regions.

As shown in Figure 7a and the enlarged section, a pronounced accumulation of coalesced crystals is observable around the grain edges, where hollow and indentation surfaces are present. Experimental observations indicate that spatial locations characterized by roughness and surface irregularities host more mineral nucleation and subsequent crystal growth. Under similar physicochemical conditions, these preferential sites are formed due to differences in surface characteristics manifested in surface free energy and increased surface–interface potential to attract solid formation and growth.

The experimental observations showed that substrate composition and substrate surface free energy influence nucleation and precipitation of crystals in multi-mineral heterogamous systems. In particular, at lower Ω values where the impact of preferences is pronounced, one can observe that certain mineral phases were the winning substrate in attracting more crystals compared to others. For instance, as shown in the mineral phase maps in Figure 7, in our system, the quartz grains were comparatively more favorable locations than feldspars after the three favored spots listed above. Silica (Si) elemental phase map representing quartz (Figure 7c) exhibits more solid accumulations (dark spots) compared to that of aluminum (Al) and sodium (Na) representing feldspar grains.

The experimental observations on the impact of primary and secondary substrates highlight the importance of implementing favorable nucleation and growth sites in the RTM of heterogeneous porous media. It is of paramount importance to identify, delineate, and predict where nucleation and precipitation start under different boundary conditions. Without proper characterization of the spatial location of preferential crystallization and accumulation sites, the theoretical models aiming to describe flow and transport in natural porous media cannot represent realistically the physics and processes occurring in various geo-environmental applications.

## 3 Implications for Thermo–Hydro–Mechanical–Chemical Processes

Flow and transport of a chemically reactive fluid through permeable porous medium encompass several classes of fluid–rock interactions. In many engineering processes or natural systems in the environment, precipitation and dissolution reactions are of primary importance because of their significant influence on the pore space geometry, fluid percolation pathways, and mobility of solute and aqueous ions. For example, contaminant transport away from waste disposal sites, radionuclide migration from radioactive waste repositories, geological CO_2_ sequestration, saline water evaporation from soil affecting land–atmosphere interactions, hydrothermal circulation, and in other areas such as sediment diagenesis, ore body formation, preservation of building materials, and petroleum reservoir stimulation. Chemical interactions and disequilibria induced by reactive transport often alter the fluid flow, deformation and geomechanics, and heat flow, which together shape the coupled thermo–hydro–mechanical–chemical (THMC) processes. THMC processes control the long-term fate of a fluid–rock system in geo-environments.^48,49^ Advective or diffusive transport coupled with precipitation and dissolution phenomena triggers propagation of semi-coherent concentration gradients, which may cause local buildup or reduction in solute concentrations in the aqueous and mineral phases.^5,40,50−52^

Mineral nucleation, precipitation, and growth might be considered a blessing or a threat depending on the system and process in which it takes place.^53^ For instance, in geological CO_2_ storage, precipitation can occur in fracture networks that can serve as the primary fluid flow conduit inside reservoirs^54,55^ or may serve as leakage pathways inside caprock units.^2,42^ It can partially or entirely clog pores and throat in the reservoir rock.^3,32,34,35,56,57^ Moreover, it can seal cracks and fractures inside well-casing cement.^58^ Therefore, mineral precipitation can be undesired when it negatively affects pore space geometry via deteriorating injectivity and storage capacity. On the other hand, it can be enormously advantageous when mineral formation prevents or minimizes CO_2_ migration and leakage. Mineral nucleation and precipitation change the porous medium’s hydrodynamics in all these scenarios, including absolute and effective permeability. Changes in permeability, in turn, translates into the alteration in fluid flow and transport of solutes, chemical species, and reaction rates.

The present study focuses on surface mineral nucleation, precipitation, and growth. We have shown that nucleation is the first step in the precipitation and growth chain of events. The experimental results indicated the probabilistic nature of the process, which is affected by the physiochemistry of the aqueous phase and governed by fluid–solid surface interactions. The research outcomes highlighted that it is crucial to consider surface properties, preferential locations, and nucleation and growth on the previously precipitated crystal (we called it secondary substrate in this paper). Understanding the consequences of mineral nucleation and growth within the porous medium across spatiotemporal scales and the fate of fluid flow and solute transport requires knowledge of pore space geometries, composition, and surface properties. The RTM attempts for precise and realistic predictions need to take into account these aspects. We also underscore the importance of theoretical reactive transport models considering the nucleation events before crystal formation.^5,7,59,60^ Nucleation events, which are probabilistic,^5,7,32,61−63^ occur only after a specific induction time and when species concentrations reach a certain threshold. During a nucleation event, a crystal nucleus forms from a supersaturated aqueous solution. Then, it undergoes processes of stabilization, growth, ripening, phase transformation, and crystallization.^64^ The nucleus may form in the bulk of a fluid called homogeneous nucleation or form at the solid–liquid interface called heterogeneous nucleation. In the present research, we provided evidence of probabilistic heterogeneous nucleation under various boundary conditions. The results showed that probabilistic nucleation contributes to broad stochastic distributions in both amounts and locations of crystals in temporal and spatial domains.

## 4 Materials and Methods

### 4.1 Sandstone Substrates and Stock Solutions

We chose a natural multi-mineral quartz-rich sandstone as the substrate. The Brumunddal eolian and fluvial red sandstones (formally Mauset formation) were deposited north of the Oslo rift at the beginning of the Permian period.^36^ It shows a good reservoir quality with an average porosity of 18% (15–24%) and permeability of 100 mD (50–200 mD).^37^ We cut core samples of the Brumunddal sandstone in disk shape with 2.5 cm diameter and 1.5 cm height to prepare substrates for microfluidic experiments. First, the disk shape specimens were washed in de-ionized water (DI-water) and an ultrasonic bath to clean the substrate surfaces and sandstone porous medium. Subsequently, a series of automatic grinding and polishing steps were conducted to provide a flat unscratched surface. After polishing, cleaning in an ultrasonic bath was repeated to remove the fragments from the surface.

For the calcite formation experiments, we prepared stock solutions from respective crystalline solids (ACS reagent, ≥ 99.8%) of calcium chloride (CaCl_2_) and sodium bicarbonate (NaHCO_3_) by adding the well-defined weight of salts to the DI-water (Milli-Q water), as presented in Table 1. We used the PHREEQC v3 package^38^ for aqueous geochemical calculations to compute solute supersaturation before the experiments. The supersaturation (Ω) is defined as the corresponding saturation ratio given by the ion activity product divided by the equilibrium constant. The stoichiometry estimates using PHREEQC were based on equilibrium with atmospheric CO_2_ pressure. As given in Table 1, the experimental fluids were prepared at three supersaturation levels, namely, 15, 50, and 130*×*.

**Table 1:** Stock Solutions Prepared for Carbonate Synthesis Experiments along with the Properties of Used Crystalline Salts

				sodium bicarbonate		calcium chloride	
	Ω^a^	pH	*C*Ca/*C*_CO_3__	molality	g/kg_w_	molality	g/kg_w_
solution I	15 (15.85)	8.8	1.54	0.005	0.42005	0.0005	0.05549
solution II	50 (47.86)	8.8	1.32	0.007	0.58807	0.001	0.11098
solution III	130 (131.83)	8.8	1.066	0.01	0.8401	0.002	0.22196

a Ω = *Q*/*K*, where *Q* and *K* indicate the ion activity product and the equilibrium constant for the reaction, respectively.

### 4.2 Laboratory Microfluidic Experiments

Nine experimental sets were conducted at three supersaturations (Ω = 15, 50, and 130*×*) and three temperatures (*T* = 20, 40, and 60 °C). For each Ω, experiments at three different temperatures were performed. The elapsed time (*t*) for the tests was 6, 48, and 96 h. Therefore, a total of 27 experiments (3 × 3 × 3 sets of Ω–*T*–*t*) were carried out. For each experimental set, two corresponding stock solutions for a given Ω (refer to Table 1) were added to the microfluidic vessel simultaneously at similar volumes to prepare 250 mL test solution. Three polished and cleaned sandstone substrates were placed inside the vessel and entirely submerged into the solution. Afterward, the microfluidic vessel was closed, sealed, and placed inside a temperature-controlled air bath to ensure temperature uniformity throughout the experiments. Furthermore, details about the laboratory apparatus are given in our previous studies.^39,40^ The reacted substrates were finally sampled out of the test vessel, washed with DI-water, and let dry overnight.

### 4.3 Solid Surface Characterization

The mineralogical composition of sandstone substrates was identified and quantified using the X-ray diffraction technique. The details are given elsewhere.^41,42^ SEM with backscattered electrons (BSE) and SE imaging was used to study the surface structure and mineral growth. SEs originate from the surface atoms and are products of inelastic interactions between the electron beam and the sample. BSEs, on the other hand, are reflected electrons after elastic interactions between the sample and the beam. SEs provide detailed information on the surface structures, whereas BSEs carry information about deeper regions and show high sensitivity to atomic numbers present on the surface. The EDS was used for chemical analyses and element mapping to identify precipitated crystals, describe the detailed mineralogy of the substrate, and delineate crystal growth patterns. A variable-pressure Hitachi SU5000 FE-SEM system (Schottky FEG) equipped with a Dual Bruker XFlash system and a high-resolution automated electron backscatter diffraction system was used to perform the SEM imaging and EDS. Carbon coating of substrates was carried out using a Cressington carbon coater (208C) to improve image quality, increase chemical analysis precision, and for better topographic examination while avoiding surface charging and potential thermal damages.

For each substrate (representing different Ω, *T*, and *t*), three random locations were selected and analyzed for SEM–EDS surface mapping. A mosaic map of nine BSE SEM images (3 × 3) was acquired at each location, covering a 3786 × 2781 μm region (approximately 10.5 mm^2^ area) with a spatial resolution of 1 μm.

### 4.4 Digital Image Processing

After several attempts to get the best precision and reproducibility, the superimposed calcium phase map (color-coded in green) on the surface mosaic map was selected as an input. Figure S1 (Supporting Information) shows a typical surface mosaic map comprising nine regions of ordinary BSE SEMs (a 3 × 3 matrix). Figure S2 (Supporting Information) presents the semi-automated workflow for digital image processing of surface mosaic maps to identify and quantify precipitated calcium carbonate crystals on the multi-mineral sandstone substrate. First, two-dimensional surface maps were filtered with a non-local means filter to remove noise and improve demarcation between phases.^43^ Subsequently, contrast enhancement filters were applied (histogram equalization and linear contrast adjustment). Based on the histogram of gray values (two distinct distributions), the mosaic maps were segmented and converted into binary images (Figure S2b). The inverted (mask) transformation of the binary map was analyzed in the ImageJ/Fiji open-access image processing package^44^ to identify, outline, and quantify the precipitated calcium carbonate crystals (Figure S2d,e).

We computed Shannon entropy for each mosaic map to quantify randomness and spatial disorder within the system. Shannon^45^ introduced the concept of entropy in information theory. The entropy of a random variable is defined as the average level of information or probability inherent in the variable’s possible outcomes. Given a random discrete variable *X*, with a likely domain of outcomes (*x*_1_, *x*_2_, ..., *x*_*n*_) and a probability of occurrence as [*P*(*x*_1_), *P*(*x*_2_), ..., *P*(*x*_*n*_)], the Shannon entropy (*E*) of variable *X* is defined as
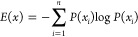
1

The Shannon entropy can also be defined as the measure of the self-information of a variable.

## Supporting Information Available

Additional details, typical high-resolution 3 × 3 SEM–EDS surface mosaic map, digital image processing workflow, probabilistic precipitation on three randomly inspected surface locations, and image processing results as a function of elapsed time (PDF)
