# Supplementary material for: Probabilistic Nucleation and Crystal Growth in Porous Medium: New Insights from Calcium Carbonate Precipitation on Primary and Secondary Substrates
Source: ACS Omega. 2021 Oct 12;6(42):28072–83. doi: 10.1021/acsomega.1c04147 (PMC8552360; doi:10.1021/acsomega.1c04147)
Supplement: Supplementary file 1 — ao1c04147_si_001.pdf [file ao1c04147_si_001.pdf]

Supporting Information

Probabilistic nucleation and crystal growth in porous medium: New insights from calcium carbonate precipitation on primary and secondary substrates

Mohammad Nooraiepour[1,*], Mohammad Masoudi[1], Nima Shokri[2] and Helge Hellevang[1]

[1] *CO_2 Storage Research Group, Department of Geosciences, University of Oslo, Norway*

[2] *Institute of Geo-Hydroinformatics, Hamburg University of Technology, Germany*

This document presents 4 Figures.

Figures S1-4 describe, respectively, a typical high-resolution 3x3 SEM-EDS surface mosaic map, digital image processing workflow, probabilistic precipitation on three randomly inspected surface locations, and the image processing results as a function of elapsed time since the onset of experiments.

[*]Corresponding author: Mohammad Nooraiepour, mohammad.nooraiepour@geo.uio.no

Digital image processing

This section presents an exemplary input surface map (*Figure S1*) and the semi-automated workflow (*Figure S2*) for digital image processing of surface mosaic maps to quantify precipitated calcium carbonate crystals on the multi-mineral sandstone substrate.

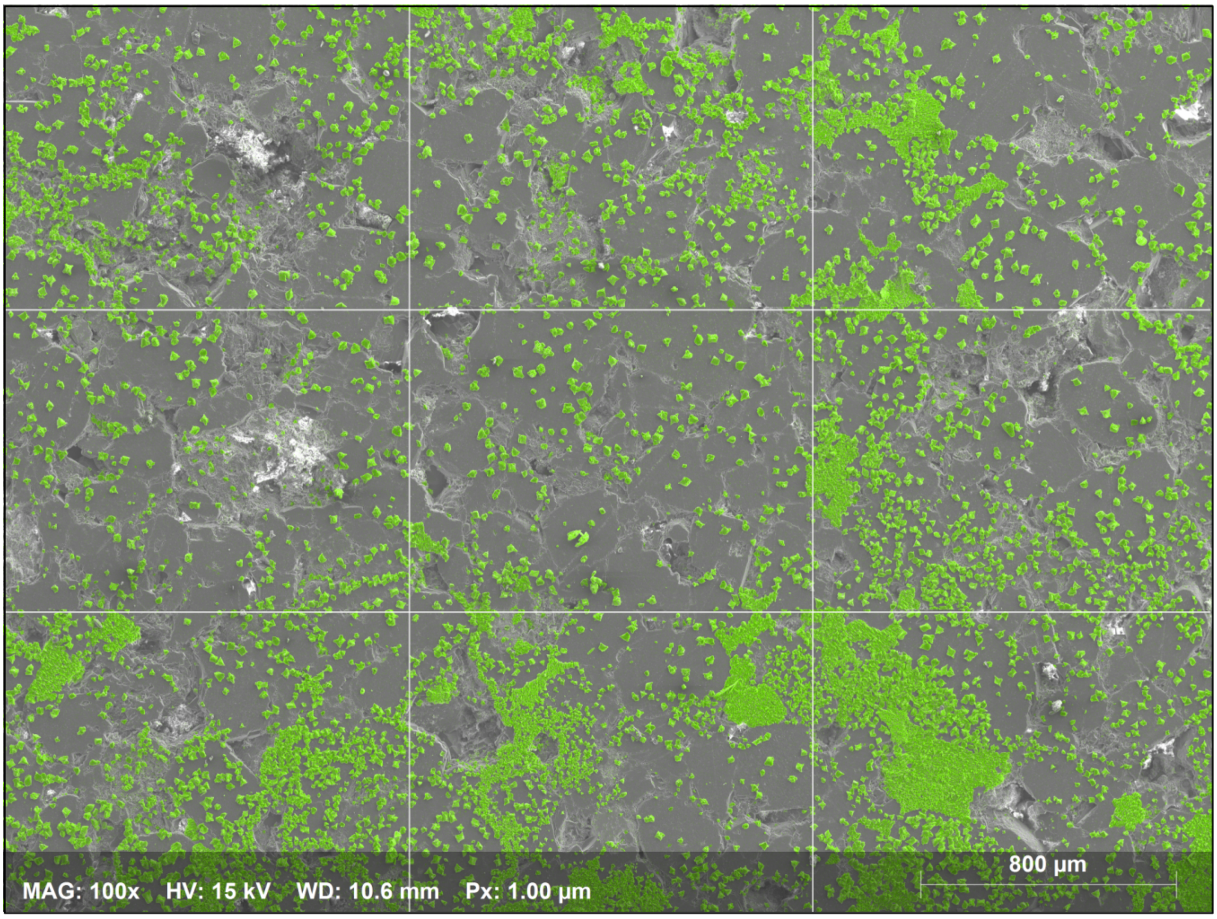

Figure S1. A typical surface mosaic map comprised of nine regions (a 3x3 matrix) of backscatter (BSE) scanning electron microscopy (SEM). The mosaic maps demonstrate the superimposed energy-dispersive x-ray spectroscopy (EDS) on top of the BSE SEM to identify calcium carbonate crystals (color-coded in green). Magnification = 100x and length-scale = 800 microns.

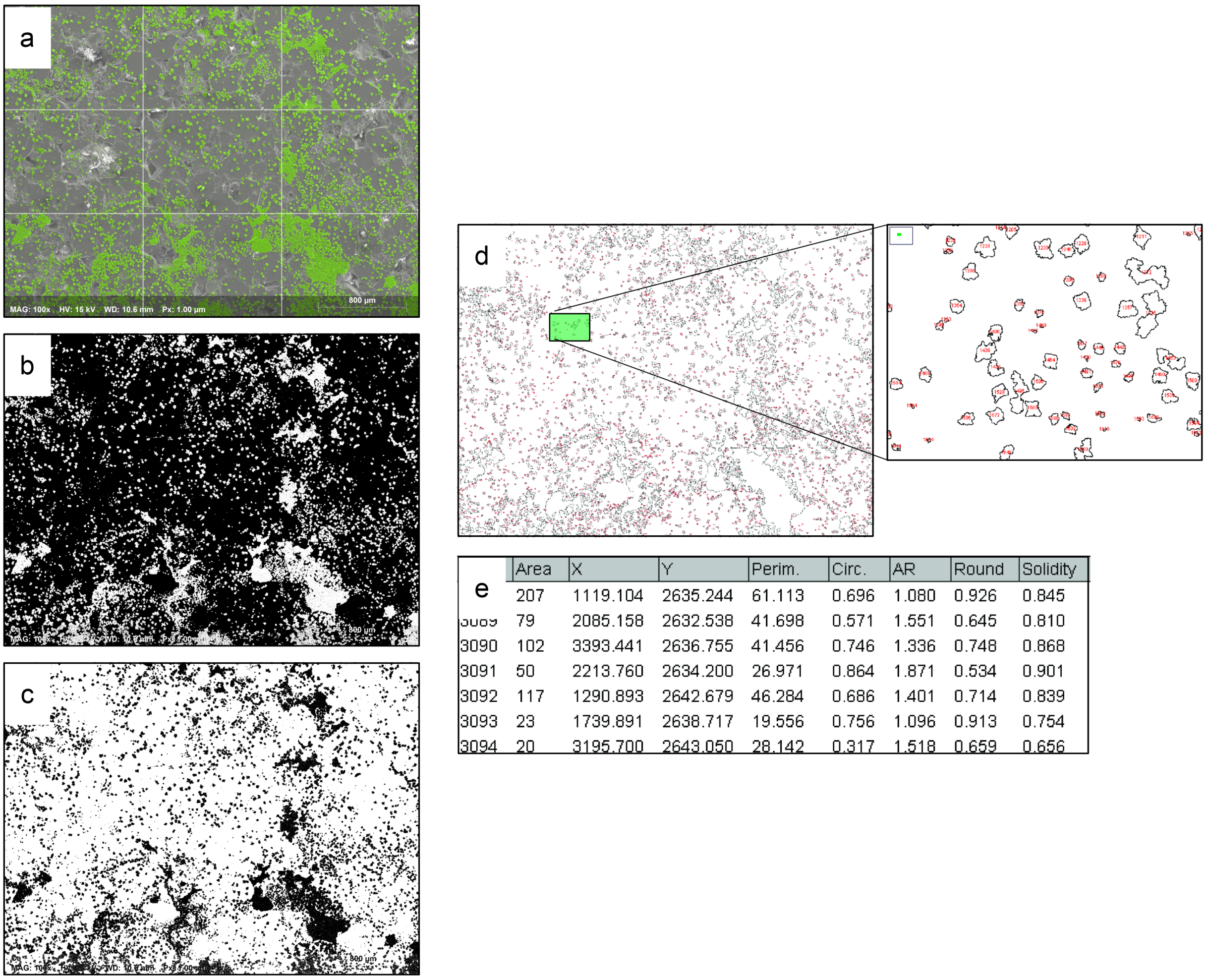

	Area	X	Y	Perim.	Circ.	AR	Round	Solidity
[illegible]	207	1119.104	2635.244	61.113	0.696	1.080	0.926	0.845
[illegible]	79	2085.158	2632.538	41.698	0.571	1.551	0.645	0.810
3090	102	3393.441	2636.755	41.456	0.746	1.336	0.748	0.868
3091	50	2213.760	2634.200	26.971	0.864	1.871	0.534	0.901
3092	117	1290.893	2642.679	46.284	0.686	1.401	0.714	0.839
3093	23	1739.891	2638.717	19.556	0.756	1.096	0.913	0.754
3094	20	3195.700	2643.050	28.142	0.317	1.518	0.659	0.656

Figure S2. Semi-automated workflow for digital image processing of surface mosaic maps to identify and quantify precipitated calcium carbonate crystals on top of the multi-mineral sandstone substrate. (a) Surface map of nine ordinary SEM-EDS micrographs acquired at each random location, where calcium phase maps are color-coded in green. (b) Binary conversion of the mosaic map in part "a" after it was subjected to noise removal, contrast enhancement, and thresholds to grayscale histogram; (c) Inverted (mask) transformation of binary map; (d) Identification and quantification of crystals using ImageJ/Fiji open-access image processing package. Every single crystal or crystal batch was assigned a number. For each number, properties of crystals were quantified considering the exact linear scale of mosaic maps and length-pixel ratio; (e) An exemplary table indicating properties of typical crystals quantified in our analysis.

Scanning electron microscopy of surface mineral growth

For each specimen, at a given supersaturation (Ω), temperature (T), and elapsed time (t), we acquired surface maps of precipitated calcium carbonate crystals at three random precipitation sites, similar to Figure S1 covering an area of approximately 10.5 mm^2. As an example, Figure S3 demonstrates such three random locations for the case $\Omega = 50x$, T = 60°C, and t = 48 hours. Figure S3 demonstrates solid deposition patterns on different spots of the heterogeneous multi-mineral sandstone substrate. This is quantified and discussed in detail in section 3.1 of the paper.

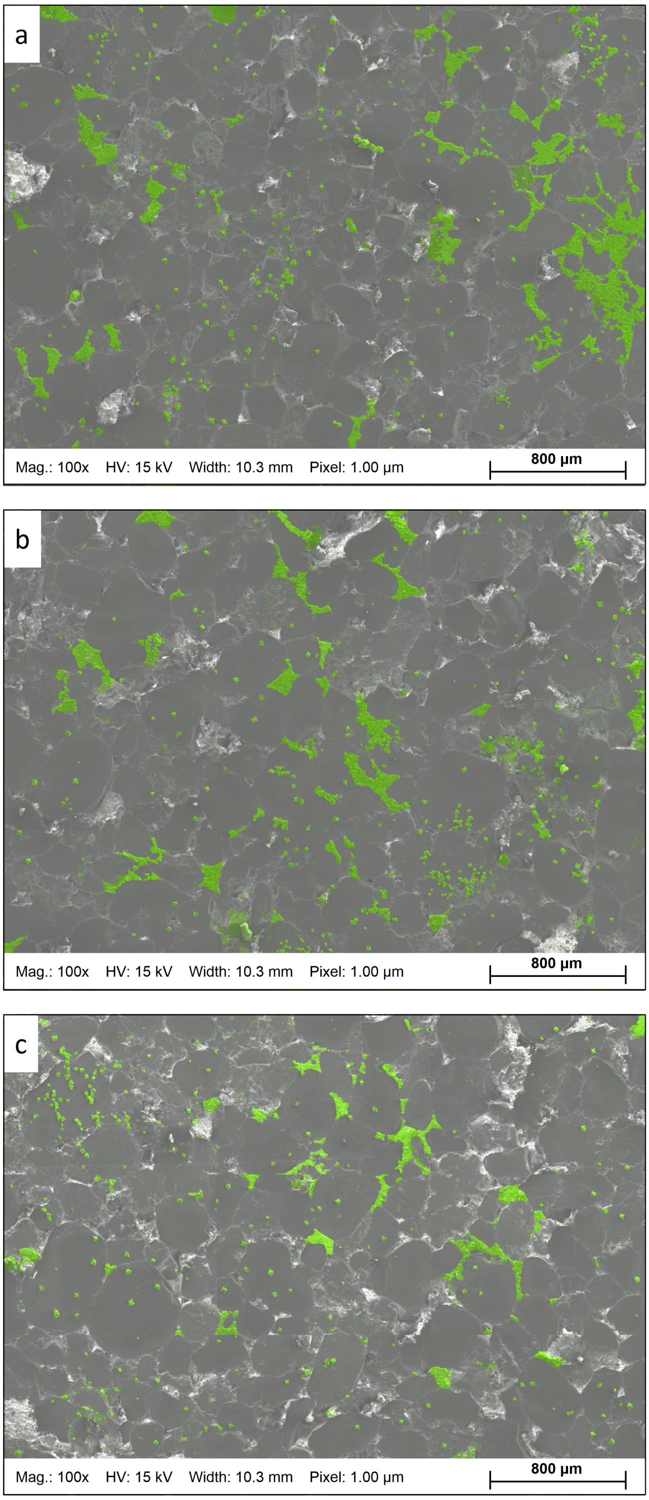

Figure S3. (a-c) SEM-EDS surface maps of three random locations inspected for the experiments at $\Omega = 50x$, T = 60°C, and t = 48 hours. It indicates the distribution and growth of crystals at randomly selected locations for a given experiment on the heterogeneous sandstone substrate. Calcium carbonate crystals are color-coded in green. Each subfigure contains nine subsections similar to Figure S1. Magnification = 100x and length-scale = 800 microns.

Image processing results

Figure S4 presents the image processing results of 27 experiments (3×3×3 sets of Ω-T-t) as a function of elapsed time. It indicates the temporal evolution of the system for different supersaturations and temperatures. Figure S4 also shows the computed entropy (E) of each mosaic map in the second ordinate (y-axis), quantifying randomness within the system.

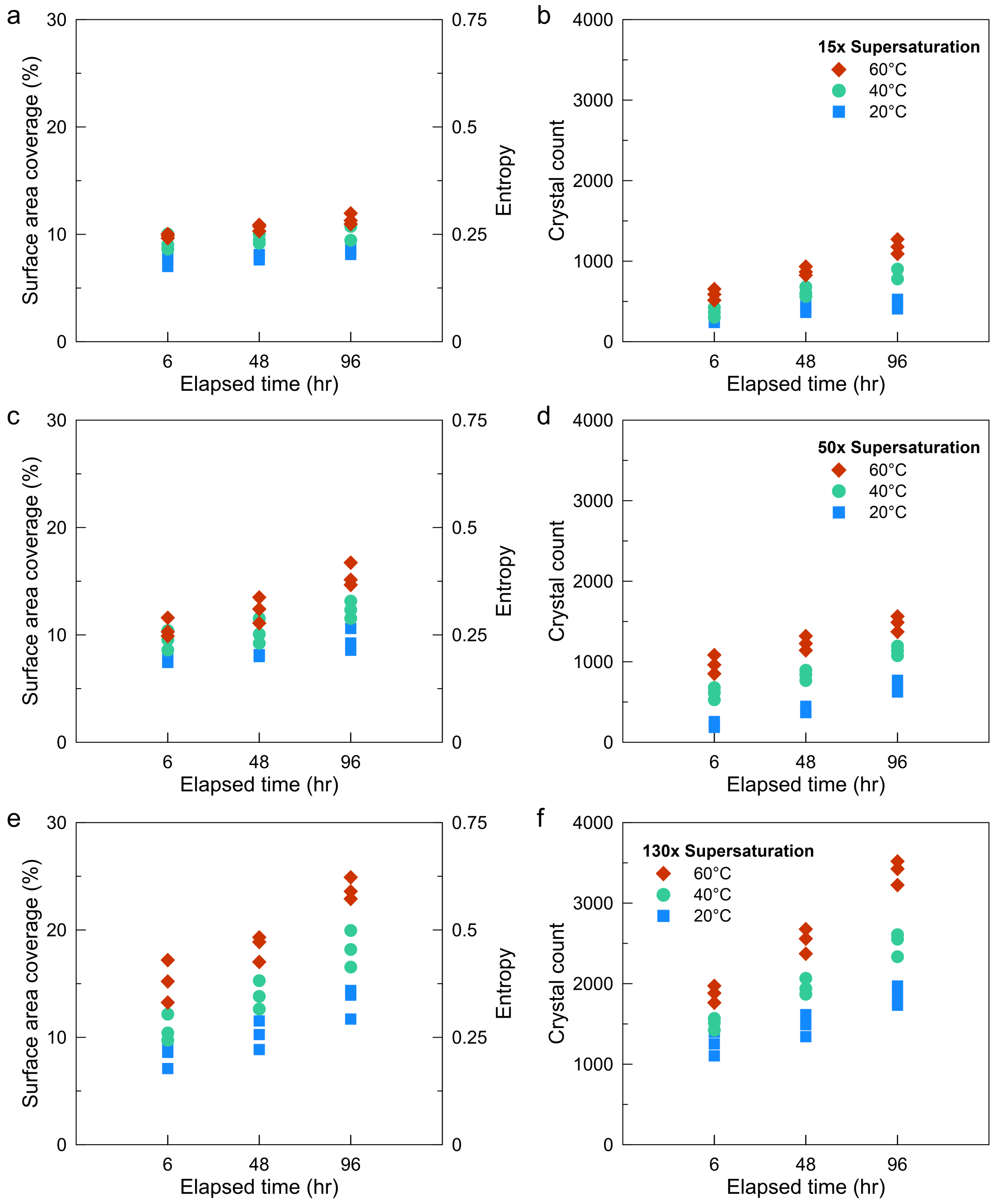

Figure S4. Total surface area coverage (%), computed Shannon entropy, and the number of the precipitated crystals derived from image processing of three random locations within each surface mosaic map. (top) 15x (middle) 50x, and (bottom) 130x supersaturation experiments as a function of elapsed time from the onset of experiments.
